# Treatment Patterns and Survival Among Veterans With De Novo Metastatic Hormone-Sensitive Prostate Cancer

**DOI:** 10.1001/jamanetworkopen.2025.9433

**Published:** 2025-05-08

**Authors:** Martin W. Schoen, Jason Doherty, Daniel Eaton, Saira Khan, Danielle Candelieri, Nicholas Fedele, Priya Baxi, Molly Wynveen, Carley Pickett, R. Jackson Wilson, Kaitlin Stackable, Kara Ingram, Krishny Karunanandaa, Rohan Agarwal, Abhinav Rajasekhar, Forest Riekhof, Srinivas Govindan, Nina Cheranda, Eric M. Knoche, Ruth D. Etzioni, R. Bruce Montgomery

**Affiliations:** 1 St Louis University School of Medicine, St Louis, Missouri; 2 Veterans Affairs St Louis Healthcare System, St Louis, Missouri; 3 Division of Public Health Sciences, Department of Surgery, Washington University in St Louis School of Medicine, St Louis, Missouri; 4 Veterans Affairs Salt Lake City Healthcare System, Salt Lake City, Utah; 5 Division of Oncology, Department of Medicine, Washington University in St Louis School of Medicine, St Louis, Missouri; 6 Fred Hutch Cancer Center, Seattle, Washington; 7 Department of Biostatistics, University of Washington School of Public Health, Seattle; 8 Division of Hematology and Oncology, University of Washington School of Medicine, Seattle; 9 Veterans Affairs Puget Sound Health Care System, Seattle, Washington

## Abstract

**Importance:**

Combination therapy for metastatic hormone-sensitive prostate cancer (mHSPC) has been widely adopted, yet clinical use and outcomes are unknown. Furthermore, optimal therapy is uncertain due to lack of direct comparison of androgen receptor pathway inhibitors (ARPIs) and docetaxel in high-volume disease.

**Objective:**

To evaluate the use of combination therapy and its association with overall survival among patients with mHSPC and to compare ARPIs vs docetaxel doublet therapy by volume of disease.

**Design, Setting, and Participants:**

This retrospective cross-sectional study was conducted in the US Veterans Health Administration among 6216 US veterans with de novo mHSPC from January 1, 2013, to December 31, 2022, treated with androgen deprivation therapy (ADT) within 3 months of diagnosis. Treatments for mHSPC were collected within 4 months of ADT. Volume of disease was assessed from radiology report review. Data were analyzed from July 2023 to October 2024.

**Main Outcomes and Measures:**

Overall survival (OS) and clinical progression-free survival (PFS), indicated by time to castration resistance or death.

**Results:**

Among 6216 male veterans with mHSPC (mean [SD] age, 73.9 [9.7] years), use of combination therapy increased from 344 of 637 veterans (54.0%) in 2020 to 465 of 737 veterans (63.1%) in 2022. Among 4106 veterans treated from 2017 to 2022, combination therapy was associated with longer OS (40.3 [95% CI, 38.0-42.1] months vs 33.0 [95% CI, 31.2-35.1] months; hazard ratio [HR], 0.80 [95% CI, 0.74-0.87]) and was used more frequently among younger veterans with fewer comorbidities. Among 1174 veterans with high-volume mHSPC, there was no difference in OS between ARPIs and docetaxel (32.3 [95% CI, 29.5-35.3] months vs 34.7 [95% CI, 31.7-37.1] months; HR, 1.06 [95% CI, 0.91-1.23]); however, ARPIs were associated with longer PFS (18.7 [95% CI, 17.1-20.9] months vs 16.0 [95% CI, 14.0-17.7] months; HR, 0.80 [95% CI, 0.70-0.91]; *P* = .001). In a multivariable model of high-volume mHSPC, there was no difference in OS between ARPIs and docetaxel (adjusted HR, 0.89 [95% CI, 0.76-1.05]). Among 366 veterans with low-volume mHSPC, there was no difference in OS between ARPIs and docetaxel (68.4 [95% CI, 52.6 months to not reached] months vs 55.3 [95% CI, 41.7-78.9] months; HR, 0.81 [95% CI, 0.58-1.13]), but ARPIs were associated with longer PFS (39.7 [95% CI, 34.3-52.9] months vs 24.0 [95% CI, 20.3-32.9] months; HR, 0.57 [95% CI, 0.43-0.76]).

**Conclusions and Relevance:**

In this cross-sectional study of veterans with de novo mHSPC, use of combination therapies increased over time and were associated with longer survival compared with ADT monotherapy. In both high- and low-volume mHSPC, no differences in OS were observed between ARPI and docetaxel combinations; however, ARPIs had longer PFS. Future research into the role of docetaxel is needed to elucidate the benefit of chemotherapy in mHSPC.

## Key Points

**Question:**

How has treatment for and survival of metastatic hormone-sensitive prostate cancer (mHSPC) changed over time?

**Findings:**

In a cross-sectional study of 6216 veterans with de novo mHSPC, the use of combination therapy increased and was associated with longer overall survival. There was no difference in overall survival for veterans with high-volume disease treated with androgen receptor pathway inhibitors (ARPIs) vs docetaxel; however, ARPIs were associated with longer progression-free survival compared with docetaxel.

**Meaning:**

This study suggests that combination therapy for mHSPC is associated with longer survival and, among patients who receive doublet therapy, docetaxel is not associated with benefit vs ARPIs.

## Teaser

This cross-sectional study evaluates the use of combination therapy and its association with survival among patients with metastatic hormone-sensitive prostate cancer and compares androgen receptor pathway inhibitors vs docetaxel doublet therapy by volume of disease.

## Introduction

Prostate cancer is increasing in incidence around the world^1^ and newly diagnosed metastatic hormone-sensitive prostate cancer (mHSPC) is common, especially in low- and middle-income countries.^2^ Metastatic hormone-sensitive prostate cancer has traditionally been treated with androgen deprivation therapy (ADT) monotherapy, but after the reporting of the CHAARTED (Chemohormonal Therapy Versus Androgen Ablation Randomized Trial for Extensive Disease in Prostate Cancer) trial in 2014^3^ and the STAMPEDE (Systemic Therapy in Advancing or Metastatic Prostate Cancer: Evaluation of Drug Efficacy) docetaxel study in 2016,^4^ combination therapy for mHSPC became standard for suitable patients. In 2017 the androgen receptor pathway inhibitor (ARPI) abiraterone acetate showed efficacy in mHSPC^5,6^; abiraterone was followed by enzalutamide^7^ and apalutamide^8^ in 2019. These combination regimens for mHSPC improved overall survival (OS) in individual trials and meta-analyses and thus are recommended for patients with adequate performance status.^9^

Overall survival in de novo (synchronous) mHSPC has been improving in the US^10^ and in European countries, and has been attributed to the use of combination therapy.^11^ However, the effectiveness of combination therapy compared with monotherapy on survival and progression among patients in general clinical practice has not been reported. Novel treatments from trials do not always translate to improvements in clinical outcomes because patients treated in the general population tend to be older, with higher burdens of comorbidities.^12^ In almost all trials of ARPIs, patients with active cardiovascular disease and low performance status were excluded, which can limit the applicability of research findings and prevent implementation in at-risk patients. Additional reports of the cardiac toxic effects of ARPIs^13^ and competing risks of death in patients with comorbidities^14^ or frail older patients^15^ could limit detection of improved survival in a nationwide clinical population.

Prior research has found that the use of combination therapy for mHSPC has increased over time. In a review of mHSPC treatment studies, combination therapy was used for approximately 30% to 40% of patients treated during 2018 and 2019.^16^ In a survey of practitioners in the US, use of combination therapy approached 59% during 2019 and 2020.^17^ In the IRONMAN (International Registry for Men with Advanced Prostate Cancer) registry that included many academic sites, the use of combination therapy for mHSPC was approximately 73%.^18^ This pattern of increasing use over time represents the typical uptake of novel therapies as they are incorporated into clinical practice, treatment pathways, or guidelines, thus allowing for reimbursement by payers. In a recent international commission on prostate cancer, the goal for combination therapy was set at 80% of new cases of mHSPC.^1^

There have been no large randomized clinical trials of docetaxel vs ARPI combination therapy, to our knowledge. Docetaxel appears to be efficacious mainly in high-volume, de novo mHSPC,^19^ while ARPIs appear to have efficacy across the spectrum of prostate cancer, albeit with greatest efficacy in high-volume, de novo disease as well.^20^ In a network meta-analyses of clinical trials, there is evidence of improved OS with an ARPI compared with docetaxel.^21^ The lack of comparisons between docetaxel and ARPIs in mHSPC make recent trials of combination therapy of ADT, ARPIs, and docetaxel difficult to interpret. In both the PEACE-1 (A Phase III Study for Patients with Metastatic Hormone-naïve Prostate Cancer) and ARASENS (ODM-201 in Addition to Standard ADT and Docetaxel in Metastatic Castration Sensitive Prostate Cancer) trials of ADT, docetaxel, and an ARPI, there was unknown benefit of docetaxel compared with an ARPI combination because it was included in both arms.^22^ An estimate of effectiveness of docetaxel vs ARPIs in a clinical population of patients with high-volume disease would help to elucidate the differences between treatments and give clinical guidance, especially for patients of borderline performance status with increased risks of toxic effects.

In situations of clinical equipoise, large data analysis from patients in an integrated care environment can identify potential differences in outcomes between therapies that are unlikely to be compared in clinical trials. Additionally, clinical analyses can provide data to understand outcomes of patients who would have been excluded from trial enrollment and report how therapeutic improvements translate to population outcomes. The Veterans Health Administration (VHA) is an ideal environment for such analyses, as patients have more equal access to care and represent a diverse population of men with prostate cancer with complete data available, including data on comorbid diseases, subsequent treatments, and OS.

## Methods

### Data and Patient Population

The VHA Informatics and Computing Infrastructure was used to access the Corporate Data Warehouse (CDW) to identify patients diagnosed with de novo (synchronous) metastatic prostate cancer as defined by the Surveillance, Epidemiology, and End Results “distant” stage from January 1, 2013, to December 31, 2022, in the Veterans Affairs (VA) cancer registry. The date of diagnosis was used as the start of observation. This cross-sectional study was approved by the St Louis Veterans Affairs Medical Center Institutional Review Board and was performed in accordance with the Declaration of Helsinki.^23^ A waiver of consent was approved given the retrospective nature of the analyses. The results are reported according to the Strengthening the Reporting of Observational Studies in Epidemiology (STROBE) reporting guideline.

From these patients, 3 cohorts were created (eFigure 1 in Supplement 1). The de novo mHSPC cohort included all patients whose initial treatment with ADT occurred within 3 months of diagnosis (referred to as the *entire cohort*) using the VHA Prostate Cancer Data Core (PCaDataCore).^24^ Combination therapy was determined as docetaxel or an ARPI (abiraterone, enzalutamide, apalutamide, or darolutamide) within 120 days of ADT. This duration was chosen for combination therapy because CHAARTED included patients who were started on docetaxel within 120 days.^3^ Docetaxel use was determined from the PCaDataCore, VA Observational Medical Outcomes Partnership, and VA Pharmacy Benefits Management. Androgen receptor pathway inhibitor prescriptions were obtained from the PCaDataCore. A second cohort was called the *modern therapy cohort* and was created to compare therapy in patients who received a diagnosis of mHSPC from 2017 to 2022 when both docetaxel and ARPIs were available. To account for immortal time bias and reduce the number of patients not eligible for therapy, all patients had to survive at least 4 months after the start of ADT.

The third cohort was called the *combination therapy with volume of disease cohort* and consisted of patients who received combination therapy with either docetaxel from July 2015 to December 2021 or an ARPI from July 2017 to December 2021. Volume of disease was assessed based on imaging reports within 4 months of diagnosis using CHAARTED criteria.^25^ Sites of disease were categorized as liver if any liver metastases were present, lung if any lung metastases were present but no liver metastases, and the remainder were considered to be bone or distant nodal metastases.

### Outcomes and Covariates

The primary outcome was OS, determined from date of diagnosis to death in the VHA Death Ascertainment File or censoring, which was July 2024. The secondary end point was clinical progression-free survival (PFS), determined as time to death or castration resistance. Castration resistance was determined from an algorithm that included natural language processing of patient records and changes in prostate-specific antigen (PSA) level.^26^ Because of known historical associations between race and ethnicity and outcomes, race and ethnicity was collected from the CDW (electronic health record) and included the categories of American Indian or Alaska Native, Asian, Black or African American, Native Hawaiian or Other Pacific Islander, White, multiple races, and unknown. Due to multiple cell sizes of fewer than 11 veterans, the categories of American Indian or Alaska Native, Asian, Native Hawaiian or Other Pacific Islander, and multiple races were combined as “other” to avoid the chance of identifying individuals. The Romano and Quan adaptations of the Charlson Comorbidity Index (CCI) and Elixhauser comorbidities were calculated based on the *International Classification of Diseases, Ninth Revision*, and *International Statistical Classification of Diseases and Related Health Problems, Tenth Revision*, codes obtained within 2 years prior to diagnosis with exclusion of prostate cancer as a variable for metastatic solid tumor.^27,28^ Second therapy including either an ARPI or a taxane (docetaxel or cabazitaxel) was determined from PCaDataCore if it was received more than 120 days after ADT. Covariates for multivariable models included age at diagnosis, race and ethnicity (Black vs other race), body mass index (BMI), PSA, and CCI.

### Statistical Analysis

Patient demographic and clinical characteristics were compared using the χ^2^ test, the *t* test, and the Kruskal-Wallis test. The Kaplan-Meier method and Cox proportional hazards regression model were used for unadjusted analyses. Multivariable Cox proportional hazards regression modeling was used to assess the association between treatments for prostate cancer, covariates, and OS. The proportional hazards assumption was tested by using a time-dependent variable as an interaction term and was nonsignificant with *P* > .05 in all models. Models were not collinear, as no variance inflation factor values were greater than 2. Missing variables were treated as a category and included in tables as “unknown.” Propensity score matching using average treatment effect on the treated weighting was performed 1:1 using a caliper of 0.01 with priority to exact matches. Data were analyzed from July 2023 to October 2024 using SPSS, version 29 (IBM Corp), and R, version 4.4.1 (R Project for Statistical Computing) was used to create figures. All tests were 2-sided and *P* < .05 was considered statistically significant.

## Results

### Cohort Description and Treatment Over Time

**Figure 1.**
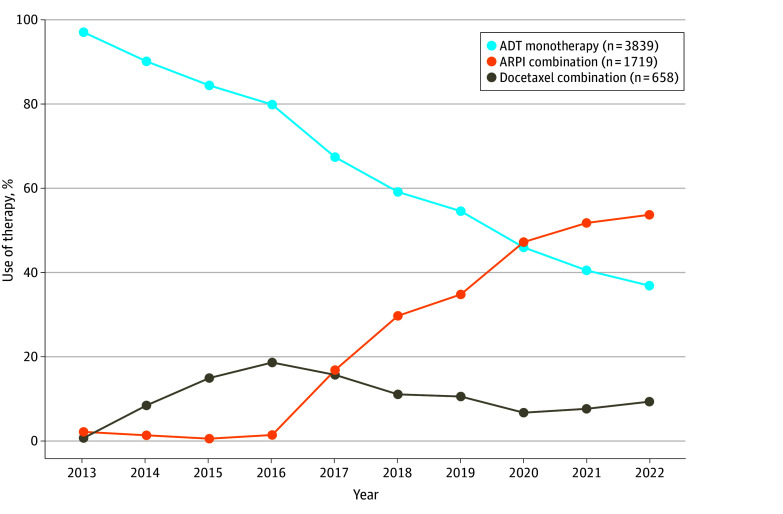
Trends in Use of Therapy for De Novo Metastatic Hormone-Sensitive Prostate Cancer From 2013 to 2022 Use of androgen deprivation therapy (ADT) monotherapy decreased from 97.1% in 2013 to 36.9% in 2022. Docetaxel combinations increased from less than 1% in 2013 to a maximum of 18.7% in 2016 to 9.4% in 2022. Androgen receptor pathway inhibitor (ARPI) combinations increased from 2.2% in 2013 to 53.7% in 2022. Overall, combination therapy increased from 2.9% to 63.1% in 2022.

Between 2013 and 2022, 6216 male veterans with de novo mHSPC who received treatment within the VHA were identified. Median year of diagnosis was 2018 (IQR, 2016-2020), mean (SD) patient age was 73.9 (9.7) years, and 1641 patients (26.4%) were Black individuals, 4118 (66.2%) were White individuals, 107 individuals (1.7%) were of other race or ethnicity, and 350 individuals (5.6%) were of unknown race or ethnicity (Table). The median CCI was 2 (IQR, 1-3). A comparison of baseline patient characteristics by treatment category is shown in the Table. Of these 6216 veterans, 3839 (61.8%) were treated with ADT monotherapy, 1719 (27.7%) with an ARPI combination, and 658 (10.6%) with a docetaxel combination. The initial ARPI was abiraterone for 1241 of 1719 patients (72.2%), enzalutamide for 408 (23.7%), apalutamide for 35 (2.0%), and darolutamide for 35 (2.0%). Figure 1 displays the treatment for de novo mHSPC from 2013 to 2022. In 2013, 399 of 411 patients (97.1%) were treated with ADT monotherapy. Starting in 2014, treatment with docetaxel increased to 37 of 437 (8.5%) and peaked in 2016 at 103 of 552 (18.7%). Use of ARPIs increased starting in 2017, with 119 of 706 veterans (16.9%) treated and continued to increase to 396 of 737 (53.7%) in 2022, the last year of inclusion. Combination therapy of either docetaxel or an ARPI with ADT became the majority of treatment in 2020, used for 344 of 637 patients (54.0%) and 465 of 737 patients (63.1%) in 2022.

**Table.** Demographic and Clinical Characteristics of US Veterans With De Novo Metastatic Hormone-Sensitive Prostate Cancer Treated Between 2013 and 2022

Characteristic	Patients, No. (%) (N = 6216)			*P* value
	ADT monotherapy (n = 3839)	ADT with ARPI (n = 1719)	ADT with docetaxel (n = 658)	
Age, mean (SD), y	75.3 (10.0)	73.3 (8.3)	66.8 (8.1)	<.001^a^
Charlson Comorbidity Index, median (IQR)^b^	2 (1-3)	2 (1-3)	1 (0-3)	<.001^c^
Elixhauser comorbidities, median (IQR)^d^	5 (3-7)	5 (3-7)	5 (3-7)	.009^c^
BMI, mean (SD)	27.4 (5.7)	28.1 (5.9)	28.6 (5.7)	<.001^a^
Race, No. (%)				
Black	967 (25.2)	457 (26.2)	217 (33.0)	.91^a^
White	2600 (67.7)	1131 (65.8)	387 (58.8)	
Other^e^	51 (1.3)	43 (2.5)	13 (2.0)	
Unknown	221 (5.8)	88 (5.8)	41 (6.2)	
PSA maximum, No. patients	3621	1637	650	.04^c^
Median (IQR), ng/mL	106.7 (33.3-378.1)	109.2 (34.8-402.1)	137.2 (40.2-544.0)	
Year of diagnosis, median (IQR)	2017 (2015-2019)	2020 (2019-2021)	2017 (2016-2020)	<.001^a^

Abbreviations: ADT, androgen deprivation therapy; ARPI, androgen receptor pathway inhibitor; BMI, body mass index (calculated as weight in kilograms divided by height in meters squared); PSA, prostate-specific antigen.

SI conversion factor: To convert PSA to micrograms per liter, multiply by 1.0.

^a^ Analysis of variance.

^b^ Range: 0 to 37, with higher scores indicating greater comorbidity.

^c^ Kruskal-Wallis test.

^d^ Range: 0 to 30, with higher scores indicating greater comorbidity.

^e^ Other race includes American Indian or Alaska Native, Asian, Native Hawaiian or Other Pacific Islander, or multiple races.

### Characteristics and Survival of Patients Diagnosed From 2017 to 2022

**Figure 2.**
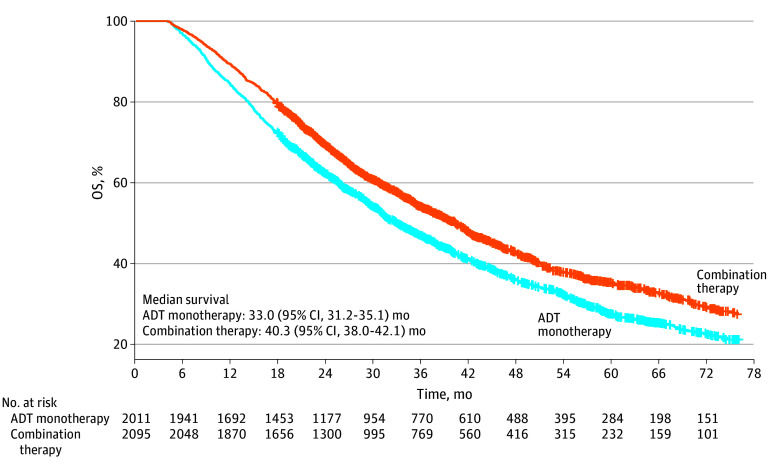
Overall Survival (OS) Among Veterans Treated for De Novo Metastatic Hormone-Sensitive Prostate Cancer From 2017 to 2022 Overall survival by treatment among 4106 patients treated from 2017 to 2022 for de novo metastatic hormone-sensitive prostate cancer who survived at least 4 months. Overall survival in combination therapy (docetaxel and androgen receptor pathway inhibitor combinations) compared with androgen deprivation therapy (ADT) monotherapy had a hazard ratio of 0.80 (95% CI, 0.74-0.87).

In the modern therapy cohort of 4106 veterans diagnosed from 2017 to 2022, those who were treated with ADT combinations were significantly younger (mean [SD] age, 72.0 [8.5] vs 76.1 [9.7] years; *P* < .001), with fewer comorbidities CCI (mean [SD] CCI, 2.0 [2.0] vs 2.5 [2.2]; *P* < .001). Survival of veterans treated with combination therapy was significantly longer than those treated with ADT monotherapy, (40.3 [95% CI, 38.0-42.1] months vs 33.0 [95% CI, 31.2-35.1] months; hazard ratio [HR], 0.80 [95% CI, 0.74-0.87]) (Figure 2). In this cohort, there was no difference in OS between ARPI combination and docetaxel combination therapy (40.4 [95% CI, 37.7-43.2] months vs 40.2 [95% CI, 36.1-44.3] months; HR, 1.06 [95% CI, 0.92-1.22]). In a multivariable model that included age, race and ethnicity, CCI, BMI, and PSA level at baseline, combination therapy was associated with a longer survival (adjusted HR [AHR], 0.89 [95% CI, 0.82-0.97]) (eTable 1 in Supplement 1).

### Volume and Site of Disease and OS

In the combination therapy with volume of disease cohort, 1540 veterans who received either docetaxel or an ARPI had imaging reports assessed for volume of disease. Veterans with high-volume disease had shorter OS than those with low volume (33.3 [95% CI, 30.9-35.7] months vs 62.5 [95% CI, 51.8-73.3 months]; HR, 0.51 [95% CI, 0.43-0.60]; *P* < .001). Veterans with high-volume disease were more likely to receive combination therapy with docetaxel (397 of 1174 [33.8%]) compared with those with low-volume disease (81 of 366 [22.1%]; *P* < .001). Among patients with high-volume disease at diagnosis, there was no difference in OS between 138 patients with lung metastases and 838 patients with bone metastases (32.2 [95% CI, 27.9-41.6] months vs 34.6 [95% CI, 31.7-37.2] months; HR, 1.00 [95% CI, 0.81-1.22]) (eFigure 2 in Supplement 1). Veterans with liver metastases (n = 71) at diagnosis had a shorter survival of 22.4 (95% CI, 13.8-30.3) months compared with high-volume bone metastases (34.6 [95% CI, 31.7-37.2] months; HR, 1.84 [95% CI, 1.43-2.37]).

### Association Between Treatment, PFS, and OS

**Figure 3.**
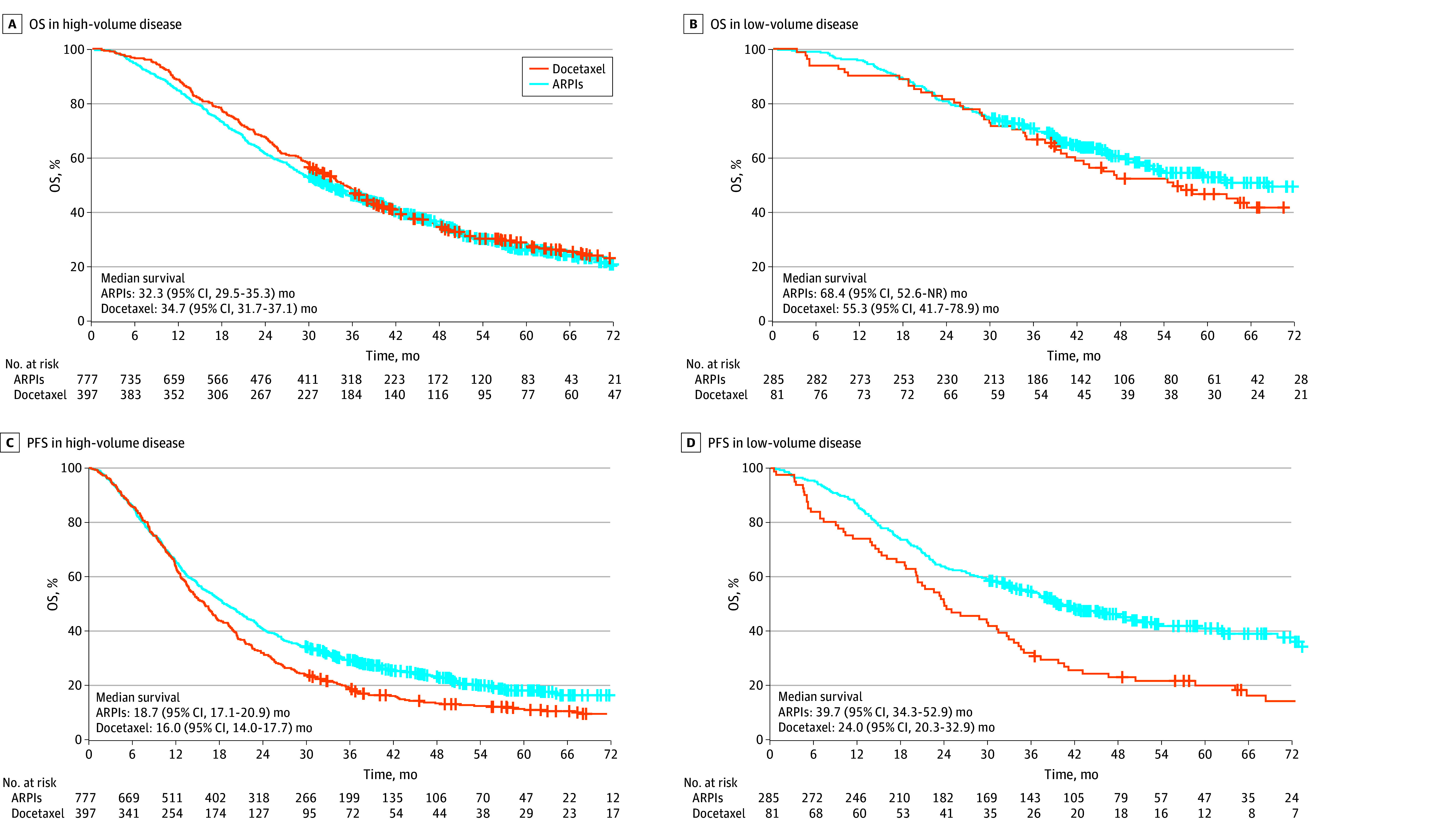
Overall Survival (OS) and Clinical Progression-Free Survival (PFS) of Veterans Treated With Combination Therapy for De Novo Metastatic Hormone-Sensitive Prostate Cancer (mHSPC) Based on Volume of Disease A, Overall survival in patients with high-volume mHSPC: hazard ratio (HR), 1.06 (95% CI, 0.91-1.23). B, Overall survival in patients with low-volume mHSPC: HR, 0.81 (95% CI, 0.58-1.13). C, PFS in high-volume mHSPC: HR, 0.80 (95% CI, 0.70-0.91). D, PFS in low-volume mHSPC: HR, 0.57 (95% CI, 0.43-0.76). ARPI indicates androgen receptor pathway inhibitor.

To assess outcomes with combination therapy, veterans in high-volume and low-volume subgroups were compared. Among 1174 veterans with high-volume mHSPC, there was no difference in OS between ARPI (n = 777) and docetaxel (n = 397) combinations (32.3 [95% CI, 29.5-35.3] months vs 34.7 [95% CI, 31.7-37.1] months; HR, 1.06 [95% CI, 0.91-1.23]) (Figure 3A). However, there was longer PFS among patients with high-volume disease treated with ARPIs compared with docetaxel combination therapy (18.7 [95% CI, 17.1-20.9] months vs 16.0 [95% CI, 14.0-17.7] months; HR, 0.80 [95% CI, 0.70-0.91]) (Figure 3C). Among 366 veterans with low-volume mHSPC, there was no difference in OS between ARPI (n = 285) and docetaxel (n = 81) combination therapy (68.4 [95% CI, 52.6 months to not reached] months vs 55.3 [95% CI, 41.7-78.9] months; HR, 0.81 [95% CI, 0.58-1.13]) (Figure 3B). However, there was a longer PFS among patients with low-volume disease treated with ARPI compared with docetaxel combination therapy (39.7 [95% CI, 34.3-52.9] months vs 24.0 [95% CI, 20.3-32.9] months; HR, 0.57 [95% CI, 0.43-0.76]) (Figure 3D).

### Multivariable and Matched Cohort Analyses

To account for differences in covariates between veterans who received docetaxel and ARPI therapy, a multivariable Cox proportional hazards regression model was created and propensity score matching was performed among veterans who received combination therapy for high-volume disease. In the multivariable model that included age, CCI, BMI, and PSA at baseline, there was no difference in OS between ARPI combinations and docetaxel (AHR, 0.89 [95% CI, 0.76-1.05]) (eTable 2 in Supplement 1). However, there was a longer PFS among patients treated with ARPI combinations compared with docetaxel (AHR, 0.74 [95% CI, 0.85-0.95]). Propensity score matching was performed, but residual differences in age were present between patients treated with docetaxel vs ARPI combinations (mean [SD] age, 67.1 [8.2] vs 68.6 [7.2] years; *P* = .01) (eTable 3 in Supplement 1). In the propensity score–matched cohort of 742 veterans, there was no difference in OS between veterans who received ARPI combinations (n = 345) vs docetaxel (n = 397) (37.4 [95% CI, 31.2-43.6] months vs 34.7 [95% CI, 31.8-37.6] months; HR, 0.91 [95% CI, 0.76-1.09]). There was longer PFS among patients treated with ARPIs compared with docetaxel (20.9 [95% CI, 17.7-24.1] months vs 16.0 [95% CI, 14.3-17.8] months; HR, 0.72 [95% CI, 0.61-0.84]).

### Subsequent Therapy

**Figure 4.**
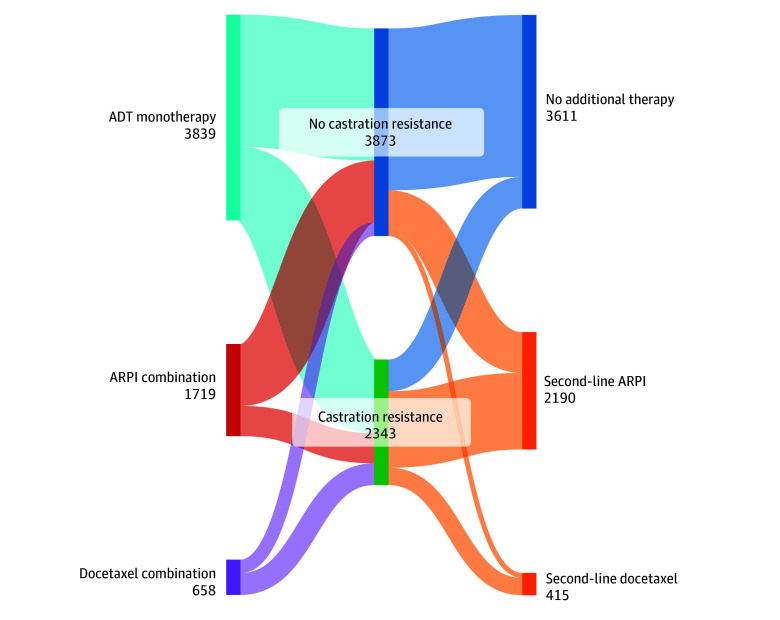
Initial and Subsequent Therapy for De Novo Metastatic Hormone-Sensitive Prostate Cancer Based on Castration Resistance Sankey diagram of initial and subsequent therapy in 6216 patients with metastatic hormone-sensitive prostate cancer in the Veterans Health Administration. There were 3873 of 6216 veterans (62.3%) who did not develop castration resistance and 3020 of 3873 (78.0%) received no further therapy. Castration resistance developed in 2343 of 6216 veterans (37.7%) and 591 of 2343 (25.2%) received no additional therapy. ADT indicates androgen deprivation therapy; and ARPI, androgen receptor pathway inhibitor.

In the entire cohort of 6216 veterans, 2343 (37.7%) developed castration resistance while 3873 (62.3%) did not, and subsequent therapy was assessed to understand patterns of treatment. Among 3839 patients initially treated with ADT alone, the immediate subsequent therapy was docetaxel for 183 (4.8%) and an ARPI for 1692 (44.1%), while 1964 patients (51.2%) received no further therapy (Figure 4). Among patients who did not receive further therapy, 1776 of 1964 (90.4%) did not develop castration resistance. Among patients initially treated with docetaxel combinations, the immediate subsequent therapy was an ARPI for 498 of 658 (75.7%) at a time to event of 477 days (95% CI, 433-521 days), with 160 of 658 (24.3%) receiving no further therapy. Among 407 patients initially treated with docetaxel who developed castration resistance, 372 (91.4%) were subsequently treated with an ARPI. Among patients initially treated with ARPI combination therapy, the immediate subsequent therapy was docetaxel for 232 of 1719 (13.5%) at a time to event of 589 days (95% CI, 513-665 days), with 1487 of 1719 (86.5%) receiving no further therapy. Among 563 patients initially treated with an ARPI who developed castration resistance, 195 (34.6%) were subsequently treated with docetaxel. Of the entire cohort of 6216 veterans, 3611 (58.1%) did not receive further therapy. Further delineation of subsequent therapy overall and among patients with castration resistance is reported in eTables 4 and 5 in Supplement 1.

## Discussion

This study found that the use of combination therapy for mHSPC has increased over time and is associated with longer survival. The increased use of combination therapy over time is expected, as implementation of novel therapies typically occurs over several years. Although ARPI combinations are appropriate for most patients with de novo mHSPC, docetaxel combination therapy was shown to be effective mainly in patients with high-volume disease in CHAARTED.^25^ Among more than 1000 veterans with high-volume disease, no difference in OS was observed with either combination. However, there was longer PFS with ARPIs compared with docetaxel. Statistical methods to account for the differences in age and comorbidities between veterans treated with docetaxel vs ARPIs showed findings similar to the overall population.

To our knowledge, there is no large randomized clinical trial of ARPIs vs docetaxel doublet combinations in mHSPC and it is unlikely that such a trial of 2 drug combinations will be performed with OS as the primary end point. The data presented here suggest that an ARPI doublet combination treatment that avoids chemotherapy is a standard of care and there is little evidence of benefit associated with a docetaxel doublet compared with an ARPI doublet. These data from clinical treatment of US veterans are supported by findings of similar studies in Europe,^29^ meta-analyses of randomized clinical trials in mHSPC,^21^ and individual patient analyses of the STOPCAP M1 (Systemic Treatment Options for Cancer of the Prostate Metastatic) collaborative that showed either trends toward longer OS with ARPIs or no difference between ARPI and docetaxel doublets.^30^ These studies also indicate longer PFS with ARPIs when reported.

Although trials of triplet combinations show that adding an ARPI to docetaxel improves OS, the role of docetaxel in addition to an ARPI is unknown, but recommended for high-volume or de novo disease that is suitable for chemotherapy.^31^ Future trials planned by cooperative groups are anticipated to investigate the role of docetaxel in the treatment of mHSPC with an ARPI doublet. This knowledge gap creates uncertainty in the role of chemotherapy or other more aggressive therapies in mHSPC, even for patients with high-volume disease.

Although there was no difference in OS, a difference in PFS was identified. Patients treated with docetaxel were more likely to progress to castration resistance compared with those treated with ARPIs after 1 year. This difference is likely due to the cessation of docetaxel after 6 cycles and the subsequent development of mCRPC. The continual therapy in ARPI combinations likely delays progression to castration resistance. More than 90% of veterans who received a docetaxel combination for mHSPC and then developed castration resistance were subsequently treated with an ARPI.

Subsequent therapy may explain the lack of difference in OS between initial docetaxel and ARPI combinations for mHSPC. However, 62.3% of patients did not develop castration resistance and 58.1% did not receive further therapy. Additionally, OS was lower in our study and the study population was older compared with clinical trials. Compared with the LATITUDE trial, in which the median patient age was 68 years, our veterans had a median age of 73 years and may have had more comorbidities and frailty than a clinical trial population. Therefore, it is likely that many veterans died from competing risks of death, such as cardiovascular disease, and never developed castration resistance and thus did not die directly from metastatic prostate cancer.

### Strengths and Limitations 

This study has several strengths, such as the use of a large, nationally representative sample of US veterans. The cohort was also limited to patients who received ADT in the VHA, which increases the accuracy of determining initial treatment of mHSPC and allows for a comprehensive dataset with a low rate of missing data in all the covariates and an accurate determination of the primary outcomes of death. Most veterans treated in the VHA system receive most, if not all, of their care within the VHA system. In addition, we used both Cox proportional hazards regression models and propensity score matching because there were important differences in characteristics of treatment groups. The use of multiple methods with similar results increased the reliability of observational methods.

This study also has some limitations. Because the US veteran population is a unique cohort of patients who have access to comprehensive care provided by the VHA system, it is uncertain if our findings are directly applicable to the nonveteran population of patients with prostate cancer or to patients who do not have similar access to care. Similar to other retrospective studies, causality between treatments for prostate cancer and mortality cannot be determined due to unknown confounders. It is likely that residual confounding influences treatment selection, such as patient functional status or aggressive or high-risk disease features and may affect the outcomes of this study.

## Conclusions

In this large, nationwide cross-sectional study of US veterans with de novo mHSPC, the use of combination therapy increased over time and reached 63.1% of veterans in 2022. Combination therapy was associated with significantly longer survival compared with ADT monotherapy. No differences in OS with either docetaxel or ARPI combinations in high- or low-volume disease were observed. There was shorter PFS with docetaxel compared with ARPI combinations in both high- and low-volume disease. Future research into the role of docetaxel in triple combination is needed to elucidate the benefit of chemotherapy in mHSPC.

## Notes

Supplement 1. **eFigure 1.** CONSORT Diagram Cohort Selection and Inclusion **eFigure 2.** Overall Survival by Site of Disease in Patients Treated With Combination Therapy **eTable 1.** Cox Proportional Hazard Model to Examine the Association of Combination Therapy With Survival Including Age, Charlson Comorbidity Index, Race, Baseline PSA and BMI in 4106 Veterans With mHSPC Diagnosed 2017-2022 **eTable 2.** Cox Proportional Hazard Model in 1174 Patients With High Volume Disease to Examine the Association of ARPI vs Docetaxel Therapy With Survival Including Age, Charlson Comorbidity Index, Baseline PSA and BMI **eTable 3.** Baseline Characteristics of the Propensity Matched Cohort of Patients Treated With Combination Therapy and Had High Volume of Disease **eTable 4.** Additional Life Prolonging Therapy Based on Initial Therapy **eTable 5.** Additional Life Prolonging Therapy Based on Initial Therapy in Patients Who Developed Castration Resistance

Supplement 2.
Data Sharing Statement
