# Supplementary material for: Treatment Patterns and Survival Among Veterans With De Novo Metastatic Hormone-Sensitive Prostate Cancer
Source: JAMA Netw Open. 2025 May 8;8(5):e259433. doi: 10.1001/jamanetworkopen.2025.9433 (PMC12062907; doi:10.1001/jamanetworkopen.2025.9433)
Supplement: Supplement 1. — eFigure 1. CONSORT Diagram Cohort Selection and Inclusion eFigure 2. Overall Survival by Site of Disease in Patients Treated With Combination Therapy eTable 1. Cox Proportional Hazard Model to Examine the Association of Combination Therapy With Survival Including Age, Charlson Comorbidity Index, Race, Baseline PSA and BMI in 4106 Veterans With mHSPC Diagnosed 2017-2022 eTable 2. Cox Proportional Hazard Model in 1174 Patients With High Volume Disease to Examine the Association of ARPI vs Docetaxel Therapy With Survival Including Age, Charlson Comorbidity Index, Baseline PSA and BMI eTable 3. Baseline Characteristics of the Propensity Matched Cohort of Patients Treated With Combination Therapy and Had High Volume of Disease eTable 4. Additional Life Prolonging Therapy Based on Initial Therapy eTable 5. Additional Life Prolonging Therapy Based on Initial Therapy in Patients Who Developed Castration Resistance [file jamanetwopen-e259433-s001.pdf]

Supplementary Online Content

Schoen MW, Doherty J, Eaton D, et al. Treatment patterns and survival among veterans with de novo metastatic hormone-sensitive prostate cancer. *JAMA Netw Open.* 2025;8(5):e259433. doi:10.1001/jamanetworkopen.2025.9433

eFigure 1. CONSORT Diagram Cohort Selection and Inclusion
eFigure 2. Overall Survival by Site of Disease in Patients Treated With Combination Therapy
eTable 1. Cox Proportional Hazard Model to Examine the Association of Combination Therapy With Survival Including Age, Charlson Comorbidity Index, Race, Baseline PSA and BMI in 4106 Veterans With mHSPC Diagnosed 2017-2022
eTable 2. Cox Proportional Hazard Model in 1174 Patients With High Volume Disease to Examine the Association of ARPI vs Docetaxel Therapy With Survival Including Age, Charlson Comorbidity Index, Baseline PSA and BMI
eTable 3. Baseline Characteristics of the Propensity Matched Cohort of Patients Treated With Combination Therapy and Had High Volume of Disease
eTable 4. Additional Life Prolonging Therapy Based on Initial Therapy
eTable 5. Additional Life Prolonging Therapy Based on Initial Therapy in Patients Who Developed Castration Resistance

This supplementary material has been provided by the authors to give readers additional information about their work.

© 2025 Schoen MW et al. *JAMA Network Open.*

eFigure 1. CONSORT Diagram Cohort Selection and Inclusion. Three analytic cohorts were created. The entire de novo mHSPC cohort included all Veterans in the VA Central Cancer Registry diagnosed with prostate cancer and SEER 'distant' stage disease from 2013-2022. These patients had to then have their initial treatment with ADT occur withing 3 months of diagnosis. The modern therapy cohort included only patient treated 2017 to 2022, therefore after ARPIs started to be available for mHSPC. The combination therapy cohort with volume of disease included patients treated with combination therapy including docetaxel from July 2015 to December 2021 or with an ARPI from July 2017 to December 2021.

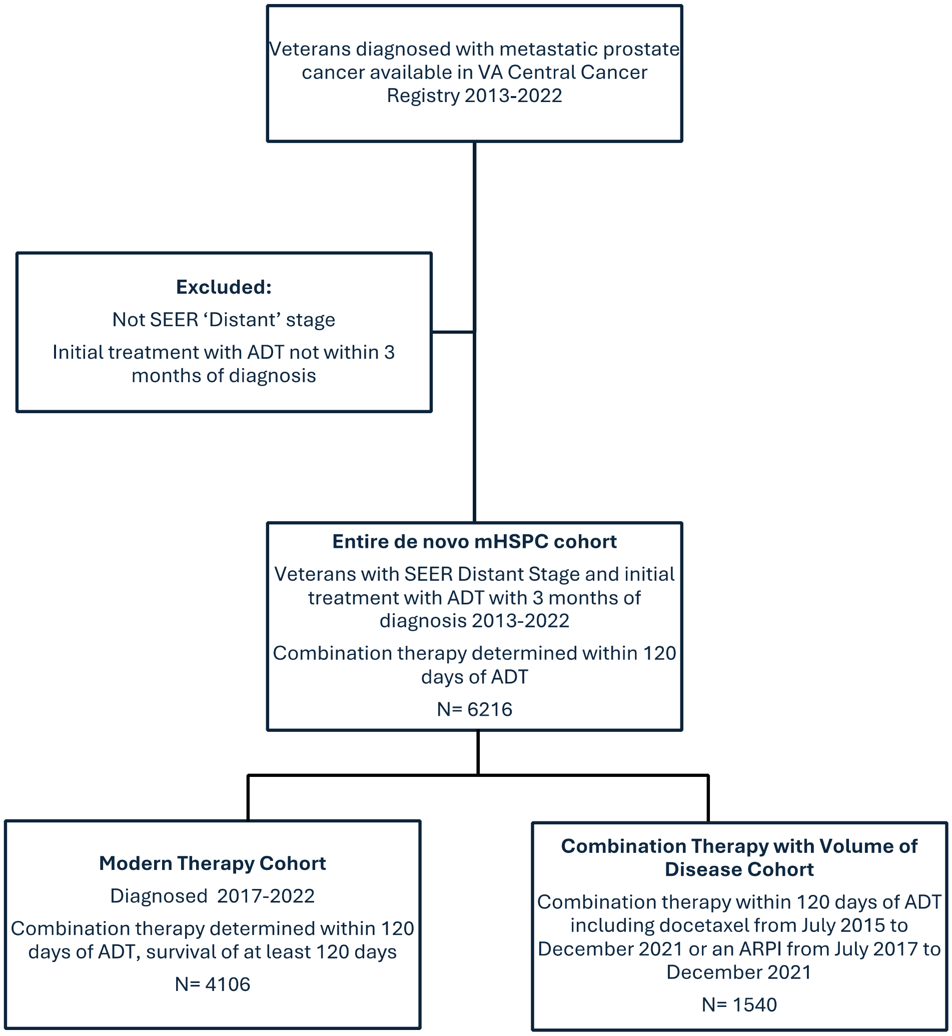

© 2025 Schoen MW et al. *JAMA Network Open.*

eFigure 2. Overall Survival by Site of Disease in Patients Treated With Combination Therapy. Low volume survival median survival was 59.2 months (95% CI, 49.9-73). High volume survival with cancer in the bones or lymph nodes but not lung or liver was 34.6 months (95% CI 31.7-37.2). In patients with lung nodules but not liver, median survival was 32.2 months (95% CI 27.9-41.6) and in patients with liver involvement, median survival was 22.4 months (95% CI 13.8-30.27).

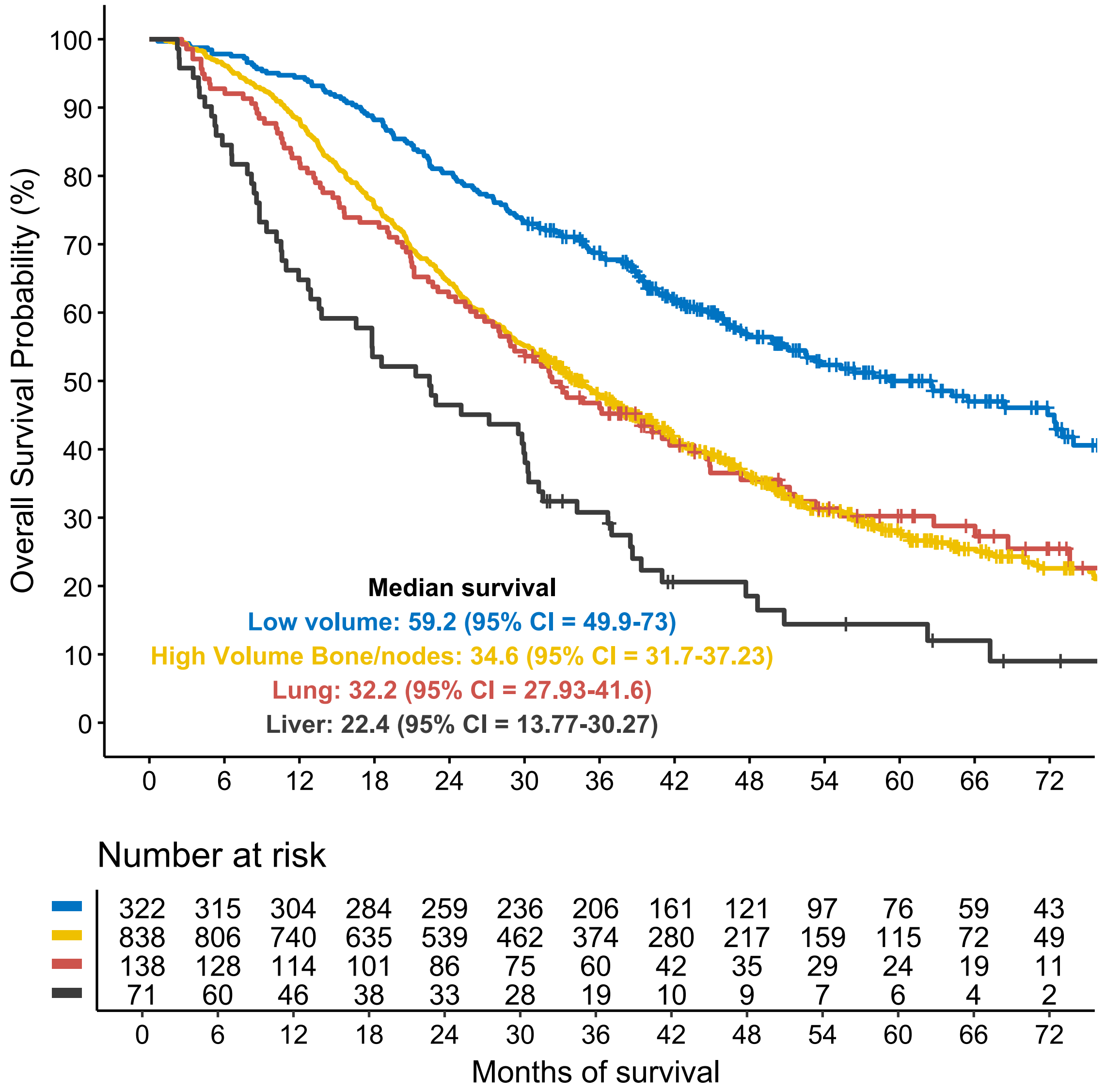

© 2025 Schoen MW et al. *JAMA Network Open.*

eTable 1. Cox Proportional Hazard Model to Examine the Association of Combination Therapy With Survival Including Age, Charlson Comorbidity Index, Race, Baseline PSA and BMI in 4106 Veterans With mHSPC Diagnosed 2017-2022

Variable	aHR#	95% CI	p
**Combination therapy vs. ADT monotherapy**	0.89	0.82-0.97	0.007
**Age: <70**	Ref		
**70-80**	1.10	0.99-1.22	0.067
**80+**	1.45	1.29-1.62	<0.001
**Charlson Index Categories: 0**	Ref		
**1**	1.17	1.03-1.35	0.017
**2-3**	1.40	1.24-1.59	<0.001
**4+**	2.30	2.02-2.61	<0.001
**Race: Non-Black**	Ref		
**Black**	0.89	0.81-0.98	0.021
**PSA Category: 0-4 ng/mL**	1.44	1.01-2.05	0.042
**4-10 ng/mL**	Ref		
**10-20 ng/mL**	1.07	0.82-1.40	0.61
**20-50 ng/mL**	1.32	1.04-1.68	0.021
**50-100 ng/mL**	1.70	1.34-2.16	<0.001
**100-200 ng/mL**	1.80	1.42-2.29	<0.001
**200+ ng/mL**	1.97	1.57-2.47	<0.001
**unknown**	1.87	1.41-2.47	<0.001
**BMI Category: < 25**	1.12	1.01-1.23	0.026
**25 ≤ BMI < 30**	Ref		
**BMI ≥ 30**	0.88	0.79-0.97	0.017

© 2025 Schoen MW et al. *JAMA Network Open.*

eTable 2. Cox Proportional Hazard Model in 1174 Patients With High Volume Disease to Examine the Association of ARPI vs Docetaxel Therapy With Survival Including Age, Charlson Comorbidity Index, Baseline PSA and BMI

Variable	aHR#	95% CI	p
**ARPI vs. docetaxel combination**	0.89	0.76-1.05	0.168
**Age (per 1 year increase)**	1.02	1.01-1.03	<0.001
**Charlson Index (per 1 point increase)**	1.13	1.09-1.18	<0.001
**PSA Category: 0-4 ng/mL**	0.88	0.44-1.76	0.71
**4-10 ng/mL**	Ref		
**10-20 ng/mL**	1.21	0.76-1.94	0.42
**20-50 ng/mL**	1.41	0.93-2.12	0.10
**50-100 ng/mL**	1.98	1.31-2.98	<.001
**100-200 ng/mL**	1.72	1.14-2.61	0.01
**200+ ng/mL**	1.76	1.20-2.58	0.004
**unknown**	1.24	0.68-2.23	0.48
**BMI Category: <25**	1.03	0.86-1.23	0.79
**25 ≤ BMI <30**	Ref		
**BMI ≥30**	1.01	0.85-1.21	0.90

© 2025 Schoen MW et al. *JAMA Network Open.*

eTable 3. Baseline Characteristics of the Propensity Matched Cohort of Patients Treated With Combination Therapy and Had High Volume of Disease

Demographic clinical characteristics	Total (n=742)		Mean difference	
	ARPI n=345	Docetaxel n=397	Cohen's d	95% Confidence Interval
**age (mean years)**	68.6	67.1	0.19	0.046 - 0.335
**Charlson Comorbidity index (mean)**	1.9	1.8	0.09	-0.051 – 0.237
**BMI (mean)**	28.2	28.7	-0.085	-0.236 – 0.065
**PSA closest to start of therapy (mean, ng/ml)**	441.7	561.4	-0.093	-0.238 – 0.052

© 2025 Schoen MW et al. *JAMA Network Open.*

eTable 4. Additional Life Prolonging Therapy Based on Initial Therapy

Subsequent Treatment	Initial Treatment		
	**ADT monotherapy n=3839 No (%)**	**ADT with ARPI n=1719 No (%)**	**ADT with docetaxel n=658 No (%)**
**Abiraterone**	1692 (44.1)	92 (5.4)	367 (55.8)
**Enzalutamide**	1151 (30.0)	332 (19.3)	269 (40.9)
**Apalutamide**	43 (1.1)	58 (3.4)	6 (0.9)
**Darolutamide**	69 (1.8)	33 (1.9)	47 (7.1)
**Docetaxel**	299 (7.8)	193 (11.2)	-
**Cabazitaxel**	132 (3.4)	70 (4.1)	134 (20.4)
**Sipuleucel-T**	<11 (-)	<11 (-)	<11 (-)
**Radium-223**	89 (2.3)	44 (2.6)	45 (6.8)
**Olaparib**	50 (1.3)	45 (2.6)	26 (4.0)

© 2025 Schoen MW et al. *JAMA Network Open.*

eTable 5. Additional Life Prolonging Therapy Based on Initial Therapy in Patients Who Developed Castration Resistance

**Subsequent Treatment**	**Initial Treatment**		
	**ADT monotherapy n=1373 No (%)**	**ADT with ARPI n=563 No (%)**	**ADT with docetaxel n=407 No (%)**
**Abiraterone**	686 (49.9)	50 (8.9)	243 (55.8)
**Enzalutamide**	458 (33.3)	223 (39.6)	113 (40.9)
**Apalutamide**	<11 (-)	12 (2.1)	<11 (-)
**Darolutamide**	16 (1.7)	32 (56.8)	13 (7.1)
**Docetaxel**	250 (18.2)	159 (28.2)	-
**Cabazitaxel**	118 (8.6)	64 (11.4)	121 (29.7)
**Sipuleucel-T**	<11 (-)	<11 (-)	<11 (-)
**Radium-223**	70 (5.1)	34 (6.0)	42 (10.3)
**Olaparib**	31 (2.3)	36 (6.4)	25 (6.1)

© 2025 Schoen MW et al. *JAMA Network Open.*
