# Supplementary material for: Treatment Patterns and Survival Among Veterans With De Novo Metastatic Hormone-Sensitive Prostate Cancer
Source: JAMA Netw Open. 2025 May 8;8(5):e259433. doi: 10.1001/jamanetworkopen.2025.9433 (PMC12062907; doi:10.1001/jamanetworkopen.2025.9433)
Supplement: Supplement 2. — Data Sharing Statement [file jamanetwopen-e259433-s002.pdf]

# Data Sharing Statement

Schoen. Treatment Patterns and Survival Among Veterans With De Novo Metastatic Hormone-Sensitive Prostate Cancer. *JAMA Netw Open*. Published May 08, 2025.
doi:10.1001/jamanetworkopen.2025.9433

**Data**
**Data available:** Yes
**Data types:** Deidentified participant data, Other (please specify)
**Additional Information:** De-identified participant data is available upon request to the first author and subject to the execution of a data use agreement for a specified research purpose.
**How to access data:** Martin.schoen@va.gov
**When available:** With publication

**Supporting Documents**
**Document types:** None

**Additional Information**
**Who can access the data:** Researchers whose proposed use has been approved via a data CRADA
**Types of analyses:** Proposed use has been approved via a data CRADA
**Mechanisms of data availability:** With signed data access agreement
